# Genetic Research: Selfish DNA versus Vector-Borne Disease

**Published:** 2008-02

**Authors:** Ernie Hood

Malaria, carried by *Anopheles* mosquitoes, infects up to 500 million people each year and kills 1 million, most of them children in sub-Saharan Africa. Dengue fever, a viral disease transmitted by *Aedes* mosquitoes, is now endemic in more than 100 countries and strikes an estimated 50 million people per year. Major international efforts to control, prevent, or eradicate these diseases are in place, but traditional methods such as vaccines and insecticides have met with limited success. Now scientists are exploring the use of so-called selfish DNA to bioengineer mosquitoes that will take over vector populations, eventually suppressing the diseases altogether.

Selfish DNA is defined as a segment of the genome with no apparent function other than to ensure its own replication. Such elements—which include transposons and homing endonuclease genes—are unique in that they can replicate themselves within a genome and are not necessary for the reproductive success (or “fitness,” in evolutionary terms) of the host organism. Selfish genes may be the ideal vehicles to deliver knockout blows to vector-borne diseases.

“You can do this in two ways,” says Fred Gould, a professor of entomology at North Carolina State University. “One is to have the selfish DNA element be neutral—that is, it just inserts itself into the population and doesn’t actually lower the fitness of the organism, but it carries with it . . . an antipathogen gene. So you wind up with a population that looks the same and has the same fitness, but can’t transmit the disease. The other approach is to actually have these selfish genes drive through a [vector] population and decrease its fitness, so that their density goes down.”

Some of these methods have shown success in the laboratory, but they are all still many years away from field deployment. Hoping to accelerate that process, Gould and 2 colleagues organized a conference held 5–7 December 2007 at the National Evolutionary Synthesis Center (NESCent) in Durham, North Carolina. Part of NESCent’s mission is to host “catalysis meetings”—intensive interactive sessions that bring together diverse groups to spark new ideas and new collaborations in emerging fields.

“Selfish DNA and the Genetic Control of Vector-Borne Diseases” gathered approximately 30 researchers whose work ranges from basic to applied science, and whose focus ranges from the molecular to the population level. “Having people with really specific expertise in the basic sciences talking to people who are trying to apply these things gave good insights,” says Gould. “There was a deep interdisciplinary interaction, and it wasn’t just superficial or lip service.”

Participants discussed some of the enormous challenges that remain to be overcome before any of these strategies will be ready for large-scale deployment. Among these are perfecting the models and determining the optimal combination of gene drive systems (i.e., methods of effectively introducing the desired gene into the population) and effector genes (which encode the antipathogenic element) to maximize vector control and minimize the development of resistance by the pathogens.

So when might we realistically expect to see mosquitoes modified with selfish DNA deployed in the field? “We’ve been saying ten years for the last ten years,” says Anthony James, a professor of microbiology and molecular genetics at the University of California, Irvine, and principal investigator for a large, multicenter research project into several of the applications being developed for genetic control of dengue virus transmission. “It’s such an adjustable horizon because as we get closer to something actually working, the size and scope of the challenges actually change.” The risk, says Gould, is that a prematurely deployed system would fail to control the disease, resulting in a rebound effect by resistant disease strains and perhaps permanently closing the window on all genetic control strategies.

Another question is whether the public can—or indeed should—accept the idea of releasing genetically modified organisms into the environment, let alone insects that are encouraged to spread, invade, and supersede native populations. Says Gould, “I think it’s important to stress that there are a million children a year dying of malaria, and preventing that is a much bigger benefit than the risk of a single gene moving into another *Anopheles* species—the risks of genes moving and disrupting habitats are small.”

## Figures and Tables

**Figure:**
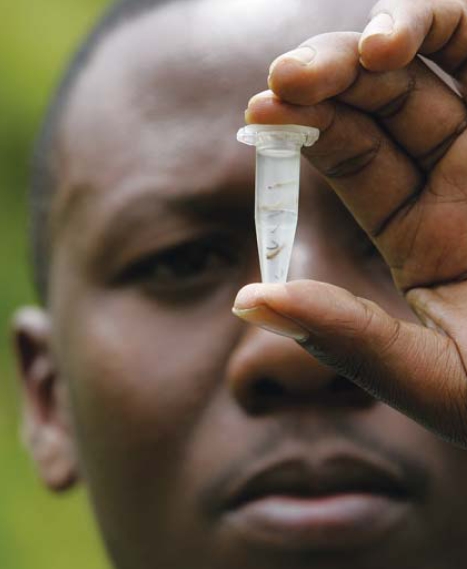
Mosquito larvae to be tested for malaria, Kenya

